# Non-magnetic four-port electronic circulators based on $90^\circ$ non-reciprocal phase-shifters

**DOI:** 10.1038/s41598-024-54468-0

**Published:** 2024-02-18

**Authors:** Dror Regev, Shaked Regev, Shimi Shilo, Doron Ezri, Nimrod Ginzberg, Emanuel Cohen

**Affiliations:** 1 Toga Networks, Hod Hasharon, Israel; 2 Technion - Israel Institute of Technology, Haifa, Israel; 3 Stanford University, Stanford, CA USA; 4 Tel Aviv University, Tel Aviv, Israel

**Keywords:** Non-reciprocal phase shifter (NRPS), Quadrature-hybrid (QH), Electronic-circulator (EC), Four-port electronic-circulator (FPEC), Engineering, Electrical and electronic engineering, Engineering, Electrical and electronic engineering

## Abstract

This paper presents a family of four-port electronic circulators adhering to a new topology symmetry that enables linear, low-loss transistor-based circuit implementations. The underlying principle of operation employs a property of the $90^\circ$ non-reciprocal phase shifter (NRPS) derived in this article. Under quadrature excitation, the NRPS transfers or reflects exciting signals depending on their respective phase lead. The fundamental topology consists of two back-to-back quadrature hybrid couplers with a $90^\circ$ NRPS connected in parallel over the line of symmetry, interrupting the circuit’s reciprocity to achieve circular propagation by bypassing or reflecting at the NRPS but not through. We break down the circuit into three fundamental four-port sub-circuits. The transfer function of the cascaded sub-circuits enables an analysis with specific hybrid couplers. It also allows a synthesis of other four-port passive sub-circuits that, with an NRPS, achieve a four-port circulator transfer function by solving a matrix equation. Some of the mathematical solutions have circuit realizations, which are adjusted quadrature hybrid structures that differ from each other by the characteristic impedance of their arms. Two familiar solutions, including the standard quadrature hybrid and a modified design with equal $Z_0$, $\lambda /4$ arms, are simulated utilizing lossless lumped element arms and a 4-Path, 65-nm NMOS $90^\circ$ NRPS. The simulation results verify the theoretical analysis and enable a comparison between the performance of the two circuit solutions around 1 GHz. The four-port circulator with equal arms is implemented on a PCB and measured, yielding better than 1.5 dB insertion loss between the circulator ports, over 17 dB port-to-port reverse isolation, and better than 20 dBr port matching around 1 GHz.

## Introduction

Circulator RF front-end (RFFE) architectures have seen a resurgence of interest in recent years as they theoretically permit concurrent transfer of unimpaired signals between their ports at the same frequency band. Multiple path simultaneous transmission in RFFEs allows continuous advancements in communication and radar systems constrained by a single path, time-division duplex (TDD), or frequency-division duplex (FDD). Techniques such as simultaneous transmit and receive^1,2^, multiple inputs multiple outputs^3^ and carrier-aggregation^4,5^ can benefit from new multi-port, chip-scale, non-reciprocal devices that enable various paths concurrently, mitigate latency constraints, increase data rate and antenna directivity.

Research of non-reciprocal components started over seven decades ago. In 1948, Tellegen^6^ proposed a new type of a nonreciprocal, linear, two-port network element which he called the ideal gyrator. The gyrator was added to the previously known resistor, inductor, capacitor, and transformer network elements as a fifth network element. As Tellegen explained, the gyrator is a lossless nonreciprocal phase shifter that introduces a $180^\circ$ phase difference for signals propagating in opposite directions across its terminals.

Hogan^7^ proposed in 1952 a gyrator based on Faraday rotation devices in which the polarization plane of radio waves rotates due to magnetic flux resulting in different phase velocities for opposite directions.

**Figure 1:**
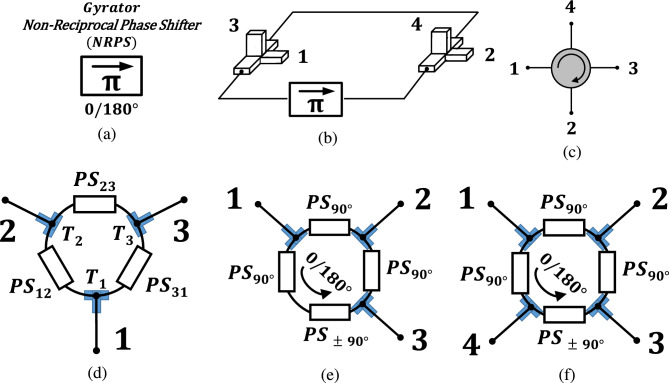
Seven decades of non-reciprocal devices: (**a**) $0/180^\circ$ gyrator—non-reciprocal phase shifter (NRPS) that introduces a $180^\circ$ phase difference for signals propagating in opposite directions. (**b**) Hogan’s 4-port magnetic circulator with two waveguide quadrature couplers and a $0/180^\circ$ gyrator. (**c**) symbol of the four-port circulator. (**d**) ring circulator^10,11^ with three T junctions and phase shifter (PS) arms where at least one PS is non-reciprocal. (**e**) ring circulator with two $90^\circ$ PS arms and a $0/180^\circ$ NRPS based on a $90^\circ$ PS in series with a $90^\circ$ NRPS^19,20^ and^21^. **f** quasi circulating quadrature hybrid (QCQH)^23^.

Furthermore, Hogan proposed the circuit symbol of the gyrator, as shown in Fig. 1a. Moreover, he was the first to describe a 4-port magnetic circulator based on two standard directional couplers and a $180^\circ$ gyrator crossing the line of symmetry, as shown in Fig. 1b. The circuit symbol he suggested is in Fig. 1c. Despite employing bulky and expensive devices, Hogan’s work pioneered the research of circulators.

In 1955, Fox^8^ distinguished between the gyrator that transfers signals from both directions and an isolator. The latter is a device that transmits signals in one direction and absorbs signals from the other. Fox also utilized transverse field effect directional phase shifters implemented by ferrite-loaded rectangular waveguides as magnetic gyrators to construct four-port magnetic circulators with a schematic similar to Hogans. In view of the tremendous interest which the four-port circulator works of Hogan, Fox, and others have started, Treuhaft^9^ analyzed circulator network properties, treated the scattering matrix as an operator, and applied cyclic substitution operations of group theory to specify the permissible topology symmetries.

A three-port ring circulator, as shown in Fig. 1d, utilizing T junctions at each port forming a triangle of phase shifter (PS) arms, where at least one phase shifter must be nonreciprocal, was proposed by Vartanian^10^ in 1956. Weiss^11^, developed a theory for synthesizing ring circulators in 1967.

Magnetic gyrators and circulators enabled new devices with non-reciprocal functionality and excellent performance. While still in use today, they are bulky and expensive and can not be integrated on-chip^12^. Hence, research on transistor-based non-reciprocal solutions became inevitable.

Transistors, biased in the active region, are nonreciprocal devices^13^, commonly modeled by a passive input network and a voltage-controlled current source at the output. They amplify signals arriving at the input direction and isolate signals from the opposite direction.

Transistor-based electronic circulator (EC) topologies have attracted researchers as early as 1965^14^. Tanaka used the intrinsic unilaterality of transistors to propose active circulators consisting of three transistors connected in a loop.

The EC designs in^14,15^, and^16^ realized compact, integrable non-magnetic circulators. On the other hand, active-based electronic circulators are limited in linearity, power handling, and noise performance. Consequently, active circulator circuits may not be suited for microwave applications that involve high-power transmissions or require high linearity and low noise figure^12,17^ including radars and transmit-receive communications, used for example, in WiFi or 5G systems.

More recently, switched transistor-based time-varying $90^\circ$ non-reciprocal phase-shifter (NRPS) N-path circuits that enable better linearity and lower noise-figure compared with active transistor-based designs were reported^18^. Ring circulators based on these N-path circuits, as shown in Fig. 1e, were implemented in^19,20^ and^21^, comprising two $90^\circ$ reciprocal and a $0/180^\circ$ NRPS. The latter utilizes a cascade of a $90^\circ$ PS and a $90^\circ$ NRPS that results in a virtual ground over the switches of the N-path NRPS for input signals at port 1, which is an advantage over the circulators of^15^ and^16^ that allow large signal swing over the NRPS and hence are less linear^22^. In^23^, a quasi-circulating quadrature hybrid (QCQH) was described similar to the circulators of^19,20^ but with a fourth port added at the other terminal of the $90^\circ$ NRPS, as shown in Fig. 1f.

A differential switch-based $90^\circ$ N-path NRPS that allows for a 3rd sub-harmonic clock and higher operation bandwidth, compared with^18^, was proposed in^24^. The principle difference between^18^ and^24^ was in the implementation and length of the delay between the path switches. Work^24^ proposed a longer delay by synchronizing back-and-forth reflections over transmission lines instead of delaying the signal over a capacitor^18^, mitigating clock leakage vulnerabilities and impairments.

A minimalistic approach for designing a sequential M-port circulator without transmission lines nor phase shifters was reported in^25^. This topology uses M-way switched capacitor paths enable a wide-band electrostatic alternative to the transmission line sections in^26^. This circuit includes only switches and capacitors, similar to two-port N-path filters^18^, but with an RC constant that allows the capacitors to charge and discharge every clock almost entirely at the adjacent port to enable only circular transmission and ensure isolation to the other ports. Nevertheless, the exponential charging and discharging of the capacitors result in a fundamental harmonic loss and non-linearity^12^.

In work^27^, a four-port circulating duplexer was proposed. It comprised a QCQH^23^ and a commercial quadrature hybrid (QH). Cascading ports 3 and 4 of a QCQH with a QH result in $4 \times 4$ S-parameters approximating a four-port circulator when implemented with an ideal $90^\circ$ NRPS^27^. However, the wide-band performance of the commercial hybrid used in^27^ degraded the overall performance by loading the N-path terminals at odd harmonics of the clock frequency^28,29^.

Chip scale gyrators inflict significantly higher losses and degraded linearity over magnetic gyrators. In the following sections, we study the properties of the $90^\circ$ gyrators and employ the results to present a new four-port circulator topology. This circuit is inspired by the devices of Hogan and Fox and the permissible symmetries of Treuhaft, yet offers a different balance that compensates for non-ideal transistor-based N-path NRPS circuits^28,29^. We start by introducing, in section II, new properties of the $90^\circ$ NRPS that enable simplified circulator design and analysis. Our generalized topology, described in section III, comprises a cascade of three four-port sub-circuits that form an equation of an ideal circulator. A rigorous study reveals a family of sixteen mathematical solutions. Realizations of the solutions are modified quadrature hybrids that differ by the characteristic impedance of their arms yet, in theory, provide the same circulator port assignment and an ideal functionality and performance. Simulations of two circulator realizations verify the analysis and indicate practical differences between the circuits.

**Figure 2:**
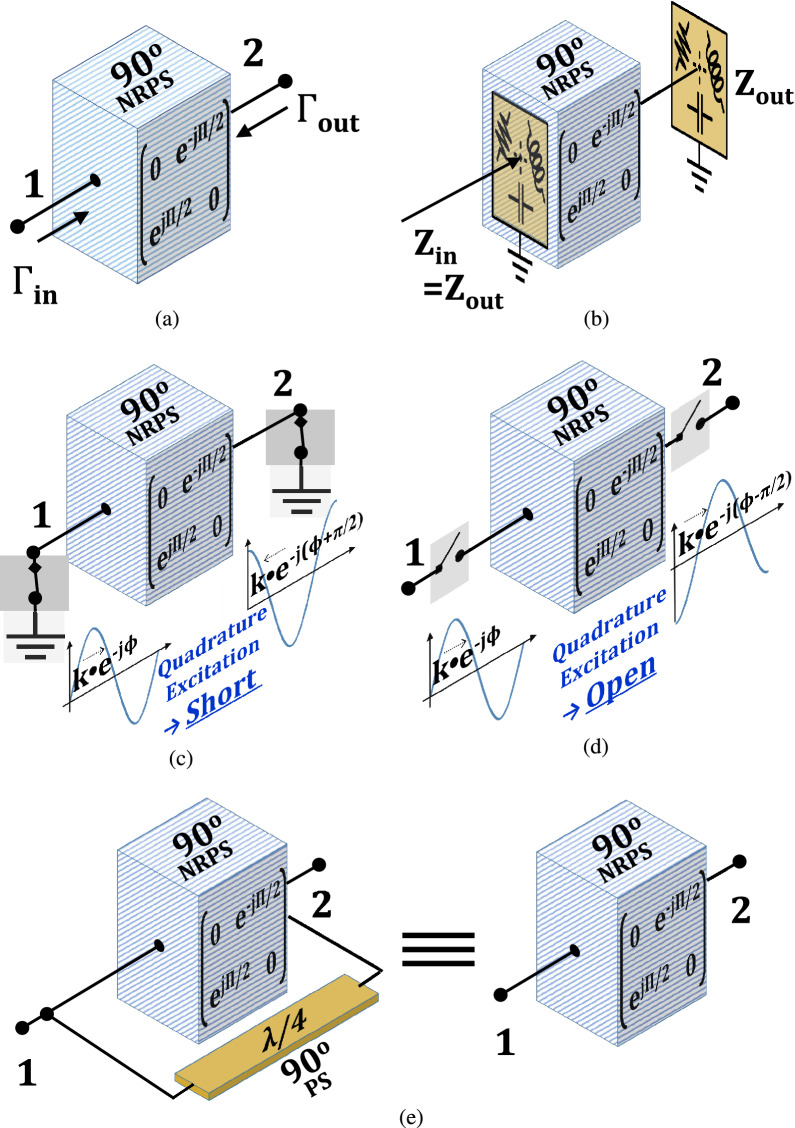
The $90^\circ$ non-reciprocal phase shifter and its network properties: (**a**) input and output reflection coefficients. (**b**) impedance transparency $\Gamma _{in} = \Gamma _{out}$ hence $Z_{in} = Z_{out}$. (**c**) quadrature excitation for phase lead of terminal 2 results in short circuit input–output effective impedance. (**d**) quadrature excitation for phase lead of terminal 1 results in open circuit input-output effective impedance. (**e**) neutralization of a $90^\circ$ transmission line load connected parallel to the $90^\circ$ NRPS.

## Network properties of the $90^\circ$ non-reciprocal phase shifter

The S-parameters of an ideal $90^\circ$ NRPS, as shown in Fig. 2a were formulated in^19,20^ and^21^ and are given as

1 $$\begin{aligned} S_\text {N90} = \left[ \begin{array}{cc} 0 &{} \quad e^{-j{\frac{\pi }{2}}} \\ e^{j{\frac{\pi }{2}}} &{} \quad 0 \\ \end{array} \right] \end{aligned}$$

### Impedance transparency

The input reflection $\Gamma _{in}$ into an ideal NRPS, where the load reflection coefficient is denoted by $\Gamma _{out}$ as shown in Fig. 2a, was given in^23^ by

2 $$\begin{aligned} \Gamma _{in} = \Gamma _{out} \end{aligned}$$

The interpretation of (2) is that ideal $90^\circ$ NRPS circuits are effectively transparent, and the input impedance equals the output impedance, as illustrated in Fig. 2b.

### Short/open reflection loading response to quadrature signal excitations

A second property of the NRPS introduced and utilized in this work describes the impedance of its terminals under quadrature excitation scenarios. The input and output reflection coefficients $\Gamma _{in}$ and $\Gamma _{out}$, as shown in Fig. 2a, are defined by the ratios between the incident power waves $a_1$, $a_2$ to the reflected power waves $b_1$, $b_2$ of the two-port network respectively^30^. Accordingly, (2) can be rewritten as

3 $$\begin{aligned} \frac{a_1}{b_1}=\Gamma _{in} ={\Gamma }_{out}=\frac{a_2}{b_2} \end{aligned}$$

Two constructive scenarios of the transparency property occur when the NRPS terminals are excited by quadrature signals when one terminal leads or lags behind the other by $90^\circ$. One can consider a quadrature excitation with a $\frac{\pi }{2}$ phase lead of the incident signal at terminal 2 over that of terminal 1, or the opposite as shown in Fig. 2c,d, respectively.

The incident quadrature waves for a $\frac{\pi }{2}$ phase lead at port 2 are denoted as $a_1=ke^{-j{\phi }}$ and $a_2=ke^{-j{(\phi +\frac{\pi }{2})}}$, where k is an arbitrary amplitude and $\phi$ is an arbitrary phase. The reflected waves at the NRPS terminals under this signal excitation are given by

4 $$\begin{aligned} \begin{pmatrix} b_1 \\ b_2 \\ \end{pmatrix} = \left[ \begin{array}{cc} 0 &{} \quad e^{-j{\frac{\pi }{2}}} \\ e^{j{\frac{\pi }{2}}} &{} \quad 0 \\ \end{array} \right] \cdot \begin{pmatrix} ke^{-j{\phi }} \\ ke^{-j{(\phi +\frac{\pi }{2})}} \\ \end{pmatrix} = \begin{pmatrix} ke^{-j{(\phi +\pi )}} \\ ke^{-j{(\phi -\frac{\pi }{2})}} \\ \end{pmatrix} \hspace{5mm} \end{aligned}$$

Hence, the output waves $b_1$, $b_2$ lag by $\pi$ ($180^\circ$) after the input waves $a_1$, $a_2$. Substituting result (4) in (3), we calculate the input and output reflection coefficients by

5 $$\begin{aligned} \Gamma _{in} =\frac{a_1}{b_1}=-1 \hspace{1mm} = \frac{a_2}{b_2}=\Gamma _{out} \end{aligned}$$

The result in (5) indicates a virtual short circuit at both terminals of the NRPS, as illustrated in Fig. 2c. Accordingly, it implies that incident quadrature signals with a phase lead at port 2 will reflect at the terminals of the NRPS.

The opposite quadrature excitation occurs when terminal 2 lags by $\frac{\pi }{2}$ behind terminal 1, as shown in Fig. 2d. The inputs, in this case, are $a_1=ke^{-j{\phi }}$ and $a_2=ke^{-j{(\phi -\frac{\pi }{2})}}$ and the respective NRPS outputs are given by

6 $$\begin{aligned} \begin{pmatrix} b_1 \\ b_2 \\ \end{pmatrix} = \left[ \begin{array}{cc} 0 &{} \quad e^{-j{\frac{\pi }{2}}} \\ e^{j{\frac{\pi }{2}}} &{} \quad 0 \\ \end{array} \right] \cdot \begin{pmatrix} ke^{-j{\phi }} \\ ke^{-j{(\phi -\frac{\pi }{2})}} \\ \end{pmatrix} \hspace{15mm}= \begin{pmatrix} ke^{-j{\phi }} \\ ke^{-j{(\phi -\frac{\pi }{2})}} \\ \end{pmatrix} \end{aligned}$$

In this case, the output waves $b_1$, $b_2$ are identical (same phase and amplitude) to the input waves $a_1$, $a_2$. Therefore, the extracted input and output reflection coefficients are

7 $$\begin{aligned} \Gamma _{in} =\frac{a_1}{b_1}=1=\frac{a_2}{b_2}=\Gamma _{out} \end{aligned}$$

Hence, for this quadrature excitation, a virtual open circuit is reflected at both terminals of the ideal $90^\circ$ NRPS, and effectively, the NRPS does not load the circuit in this case.

In conclusion, the input and output reflection coefficients of the $90^\circ$ NRPS can be inverted between short and open by reversing the excitation phase of a quadrature signal.

### Virtual neutralization of a $90^\circ$ transmission line connected across a $90^\circ$ NRPS element

We introduce here a third counterintuitive yet practical property of the $90^\circ$ NRPS. One can verify that the NRPS neutralizes the loading effect of a $90^\circ$ transmission line (TL) connected in parallel at its terminals. The proof of this theorem requires the summation of the admittance (Y) parameters of the NRPS and the TL. The Y matrix of (1) is not invertible, and hence undefined^31^. Therefore, to yield an invertible form of (1), we assign resistive losses in series with the NRPS in Fig. 2a and obtain a practical matrix $S_\text {N90-P}$ as in (14) of section V. The coefficient magnitudes of $Y_{\textrm{N90}}$, the respective non-ideal NRPS matrix, are inversely proportional to the value of the resistors added to the ports and tend to infinity when the added resistance tends to zero. Therefore, the Y coefficient magnitudes of $90^\circ$ NRPS are relatively high (as in (15) of section V) compared to the Y-parameters of the TL. Hence, the TL barely affects the overall magnitude of the parallel sum of the two elements. Effectively, the NRPS neutralizes the load of the parallel TL. This property is illustrated in Fig. 2e, and the derivation is in section V. It applies to a $90^\circ$ transmission line of any characteristic impedance, yet, naturally, for practical non-ideal NRPS circuits, the loading effect of a low-impedance TL is more significant than of a high-impedance TL.

**Figure 3:**
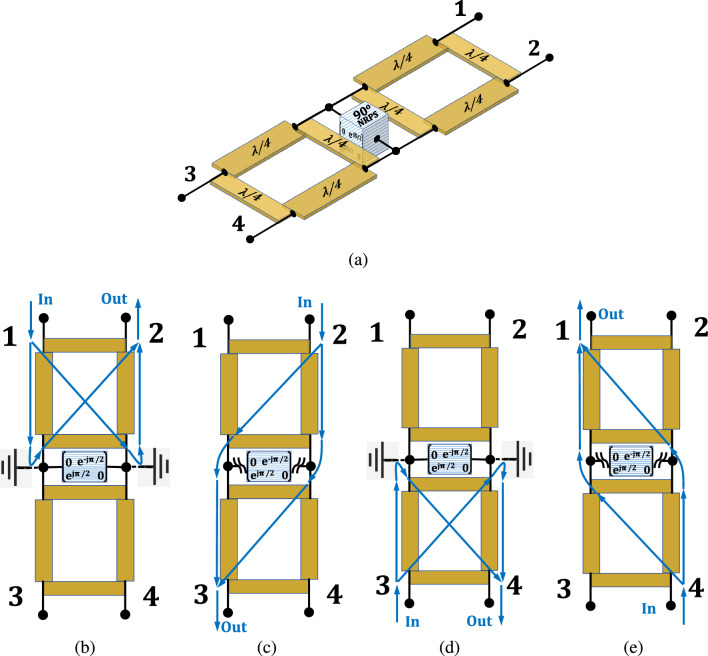
The four-port electronic circulator: (**a**) generalized schematic employing a $90^\circ$ NRPS across the line of symmetry. (**b**) signal transmission for input at port 1. (**c**) signal transmission for input at port 2. (**d**) signal transmission for input at port 3. (**e**) signal transmission for input at port 4.

**Figure 4:**
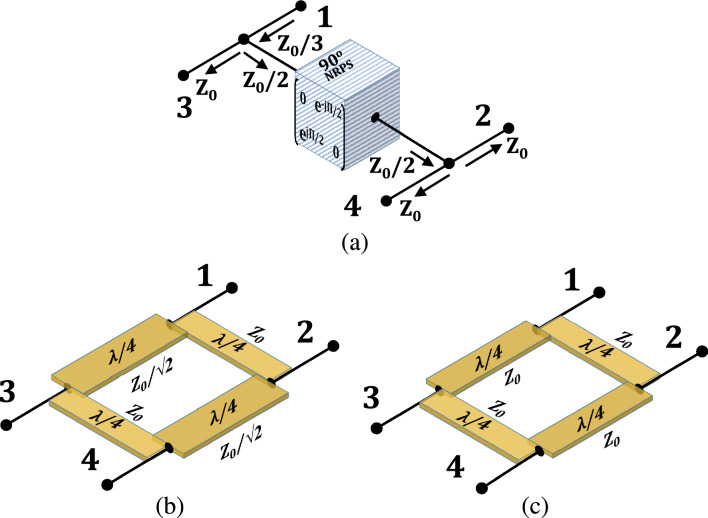
Sub-circuits of four-port circulators: (**a**) four-port presentation and analysis of the $90^\circ$ NRPS. (**b**) standard quadrature-hybrid four-arm distributed realization. **c** distributed realization of the modified quadrature-hybrids with four equal $90^\circ$ ($\lambda /4$) $Z_0$ arms.

## A four-port electronic circulator employing a $90^\circ$ non-reciprocal phase shifter across the line of symmetry

A schematic of the generalized four-port electronic circulator (FPEC) introduced in this work is in Fig. 3a. It consists of two cascaded quadrature hybrid (QH) couplers (or modified versions as will be shown) and a $90^\circ$ NRPS connected in parallel to the line of symmetry.

### Open/short analysis of the four-port electronic circulator

An intuitive analysis for the circulator with standard quadrature hybrids, shown in Fig. 4b, follows quadrature signal division properties of a QH as reflected in the S-matrix of an ideal QH^32^ given by

8 $$\begin{aligned} S_{\textrm{QH}} = - \frac{1}{\sqrt{2}} \left[ \begin{matrix} 0 &{} \quad 0 &{} \quad j &{} \quad 1 \\ 0 &{} \quad 0 &{} \quad 1 &{} \quad j \\ j &{} \quad 1 &{} \quad 0 &{} \quad 0 \\ 1 &{} \quad j &{} \quad 0 &{} \quad 0 \end{matrix} \right] \end{aligned}$$

Accordingly, the QH connected at ports 1 and 2 of the FPEC in Fig. 3a divides signals incident at port 1 equally between ports 3 and 4 of the QH, where the phase of the signal in port 4 lags by $90^\circ$ behind that of port 3. This quadrature excitation agrees with that described in (4), resulting in virtual shorts across the NRPS terminals as in (5). Therefore, the quadrature signal divided by the QH reflects entirely at the NRPS terminals. Thereafter, the signal reconstructs perfectly by the same QH at port 2 of the FPEC, as illustrated in Fig. 3b. Hence, input signals at port 1 transfer without any loss to port 2, port 1 impedance matching is perfect, and ports 3 and 4 of the FPEC are isolated from port 1.

For signals incident at port 2 of the FPEC, the NRPS terminals’ quadrature excitation is opposite from that of port 1, agrees with the phase in (6), and results in open terminals as in (7). Thus, this excitation virtually disconnects the NRPS from the circuit. In effect, the quadrature signal at the outputs of the QH connected to ports 1 and 2 reconstructs by the QH connected to ports 3 and 4 at port 3, as shown in Fig. 3c. Theoretically, the total input signal at port 2 is delivered to port 3 as if there is no NRPS in the circuit, port 2 impedance matching is perfect, and ports 1 and 4 are isolated from port 2.

The circulator symmetry ensures that input signals at port 3 reflect at the NRPS and reconstruct at port 4, as shown in Fig. 3d, whereas signals incident at port 4 divide by the QH, bypass the NRPS, and reconstruct at port 1, as shown in Fig. 3e. Accordingly, the combined 4x4 S-matrix for the FPEC, enables an ideal four-port circulator and is given by

9 $$\begin{aligned} S_{\textrm{FPEC}}= \left[ \begin{matrix} 0 &{} \quad 0 &{} \quad 0 &{} \quad j \\ -j &{} \quad 0 &{} \quad 0 &{} \quad 0 \\ 0 &{} \quad j &{} \quad 0 &{} \quad 0 \\ 0 &{} \quad 0 &{} \quad -j &{} \quad 0 \\ \end{matrix} \right] \end{aligned}$$

### Analysis by cascading the three sub-circuits of the four-port electronic circulator

An alternative derivation of (9) follows cascading properties of transfer matrices. One can analyze the FPEC schematic shown in Fig. 3a cascading three four-port sub-circuits (QH-NRPS-QH) by replacing two standard quadrature hybrids, as shown in Fig. 4b, instead of the generalized hybrids. Figure 4a illustrates the $90^\circ$ NRPS representation as a four-port circuit. Calculation of $T_{\textrm{FPEC}}$, the cascaded transfer function matrix of the FPEC, relies on the transfer (T) matrices of the QH and NRPS and is given by

10 $$\begin{aligned} T_{\textrm{FPEC}} = T_{\textrm{QH}} \cdot T_{\textrm{4PortN90}} \cdot T_{\textrm{QH}} \end{aligned}$$

where $T_{QH}$ is the transfer matrix form of $S_{QH}$ in (8)^31,33,34^ and $T_{\textrm{4PortN90}}$ is the 4-port transfer matrix of the $90^\circ$ NRPS as illustrated in Fig. 4a.

To derive $T_{\textrm{4PortN90}}$, one needs to define the 4-port S-parameters of the NRPS introduced here. The four-port formation of (1) follows utilization of the transparency property given by (2). As shown in Fig. 4a, the input impedance looking from the NRPS terminal between ports 1 and 3 into the NRPS is $Z_0/2$. The impedance seen from port 1 into the circuit is $Z_0/3$ accounting for port 3 impedance $Z_0$ connected in parallel to the impedance reflected at the NRPS terminal. A $Z_0/3$ input impedance reflects precisely a quarter of the incident power applied at port 1. The remaining three quarters are divided evenly between the equal $Z_0$ loads at ports 3, 4, and 2. Hence each port absorbs a quarter of the incident power. This explanation is also valid for incident signals at ports 2, 3, and 4. Therefore the magnitude of the reflection coefficient at all the ports is 1/2, and the sign is negative (since $Z_0/3 < Z_0$). Moreover, the magnitudes of other S-parameters are also 1/2 as each absorbs a quarter of the power. There is no phase shift between ports 1 and 3 and no phase shift between 2 and 4. The NRPS inflicts a positive $90^\circ$ phase shift between the terminal connected at ports 1 and 3 to the terminal connected at ports 2 and 4, as shown in Fig. 4a. In contrast, in the opposite direction, the phase between the terminals of the NRPS is a negative $90^\circ$. The four-port S-matrix of the ideal $90^\circ$ NRPS shown in Fig. 4a is a Hermitian matrix given by

11 $$\begin{aligned} S_{\textrm{4PortN90}} = \frac{1}{2} \left[ \begin{matrix} -1 &{} \quad -j &{} \quad 1 &{} \quad -j \\ j &{} \quad -1 &{} \quad j &{} \quad 1 \\ 1 &{} \quad -j &{} \quad -1 &{} \quad -j \\ j &{} \quad 1 &{} \quad j &{} \quad -1 \end{matrix} \right] \end{aligned}$$

Matrix (11) is not invertible; hence, its T matrix is not defined^31^. Therefore, we impose small resistive losses in series with the ports of the NRPS of Fig. 4a to find a practical NRPS S-matrix form $S_\text {4PortN90-P}$ such as (19) of section V to enable the extraction of $T_\text {4PortN90}$.

The calculation of the cascaded $T_\text {FPEC}$ follows (10), employing (8) and (19). The respective FPEC S-parameters are extracted by converting from transfer to S-parameters (T $\rightarrow$ S)^31^. The ideal $S_\text {FPEC}$ is obtained by letting the resistors added at the ports of Fig. 4a tend to zero. The result is identical to (9) as expected.

### A generalized synthesis of the four-port electronic circulator utilizing cascaded sub-circuit transfer-functions

An examination of (10) suggests that there may be multiple T solutions that satisfy $T_\text {FPEC}$ given $T_\text {4PortN90}$. To find these solutions, one must solve an equation of the form $B=X \cdot A \cdot X$. The derivation in section V reveals a family of sixteen transfer matrix solutions which, after conversion to S-parameters^33,34^, take the general form

12 $$\begin{aligned} S_{QHn}= \left[ \begin{matrix} a_n &{} \quad jb_n &{} \quad jc_n &{} \quad d_n \\ jb_n &{} \quad a_n &{} \quad d_n &{} \quad jc_n \\ jc_n &{} \quad d_n &{} \quad a_n &{} \quad jb_n \\ d_n &{} \quad jc_n &{} \quad jb_n &{} \quad a_n \end{matrix} \right] \end{aligned}$$

where $S_{QHn}$ denotes the $n^{th}$ general solution and n $\in$ 0, ... 15 with matrix (8) being one of the solutions. Another solution of interest is given by

13 $$\begin{aligned} S_{QHm} = - \frac{1}{5} \left[ \begin{matrix} -1 &{} \quad 2j &{} \quad 2j &{} \quad 4 \\ 2j &{} \quad -1 &{} \quad 4 &{} \quad 2j \\ 2j &{} \quad 4 &{} \quad -1 &{} \quad 2j \\ 4 &{} \quad 2j &{} \quad 2j &{} \quad -1 \end{matrix} \right] \end{aligned}$$

where $S_{QHm}$, denotes a modified quadrature hybrid. We found that the structure in Fig. 4c realizes (13) at the center frequency. It comprises four equal $90^\circ$ ($\lambda /4$) $Z_0$ arms and hence practical for implementation. Circuit realizations of the other solutions require further research.

**Figure 5:**
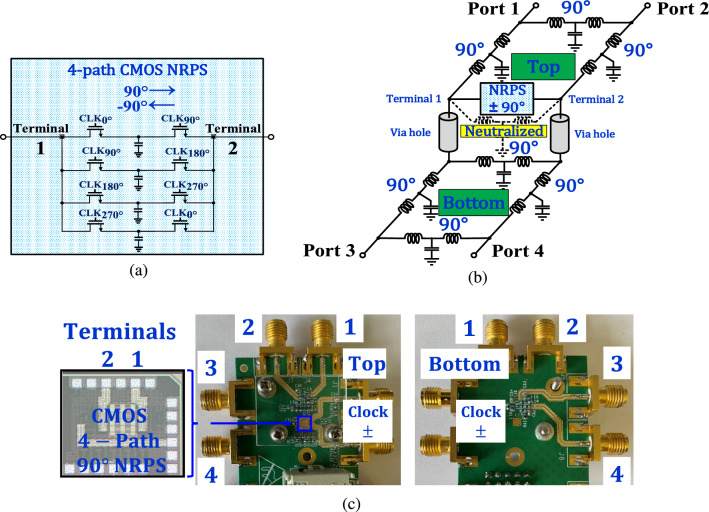
The four-port electronic circulator: (**a**) schematic of the 65 nm NMOS 4-path $90^\circ$ NRPS (**b**) the implemented circuit utilizing LCL $90^\circ$ phase shifters and a $90^\circ$ NRPS. (**c**) left - die photo, center-top side of implemented PCB, right-bottom side of implemented PCB.

### Simulation results for two four-port electronic circulator solutions based on a $90^\circ$ NMOS 4-path NRPS

We simulate the FPEC S-parameters employing solutions (8) versus (13) and given a non-ideal $90^\circ$ NRPS, such as an N-path filter. Two circuits that share the schematic, as in Fig. 5b, are compared where the first employs standard hybrids, as in Fig. 4b, and the second the modified hybrids, with equal $Z_0$ $90^\circ$ arms, as in Fig. 4c. Both FPECs employ lumped LCL (inductor-capacitor-inductor) $90^\circ$ phase shifters (impedance transformers) and a $90^\circ$ NRPS that is implemented by a 65-nm NMOS 4-Path circuit, as shown in Fig. 5a. The two-port N-path circuit was described in^18^ as a cascade of downconversion and upconversion mixers with a capacitor at base-band that serves for narrow band filtering^28,29^, centered around the clock frequency^18^. The phase shifting property is attributed to, and can be controlled by, the delay between the clocks of the downconverter and the upconverter^18^. It can be set as a quarter of the clock period, hence $90^\circ$, with a polarity that depends on the N-path terminal of reference^35^. Therefore, the propagation phase reverses direction between the two terminals. In^35^, the frequency-dependent S-parameter performance of the N-path circuit was formulated.

The transmission line arms comprise ideal LCL phase-shifters (at 1 GHz) with $Z_0$ values of L = 8.7 nH and C = 2.9 pF and $Z_0/ \sqrt{2}$ values of L $=$ 6.2 nH and C $=$ 4.1 pF. The FPECs are simulated with the 4-path NRPS circuits shown in Fig. 5a, employing a 1 GHz clock frequency and a base-band capacitance of 12.5 pF. The neutralizing property of the NRPS allows the removal of one of the $90^\circ$ LCL arms in parallel with the NRPS, as shown in Fig. 5b, forming a QCQH at the top PCB. Despite the missing arm, the performance of both FPECs improves. The arm across the N-path at the bottom was not removed in order to partially compensate the NRPS parasitic^28,29^. Simulations showed that for both FPECs, one TL in parallel to the NRPS performed better than two in parallel or none, as may be possible with an ideal NRPS. The via holes were modeled by a simplified LC circuit and showed limited impact at 1 GHz.

**Figure 6:**
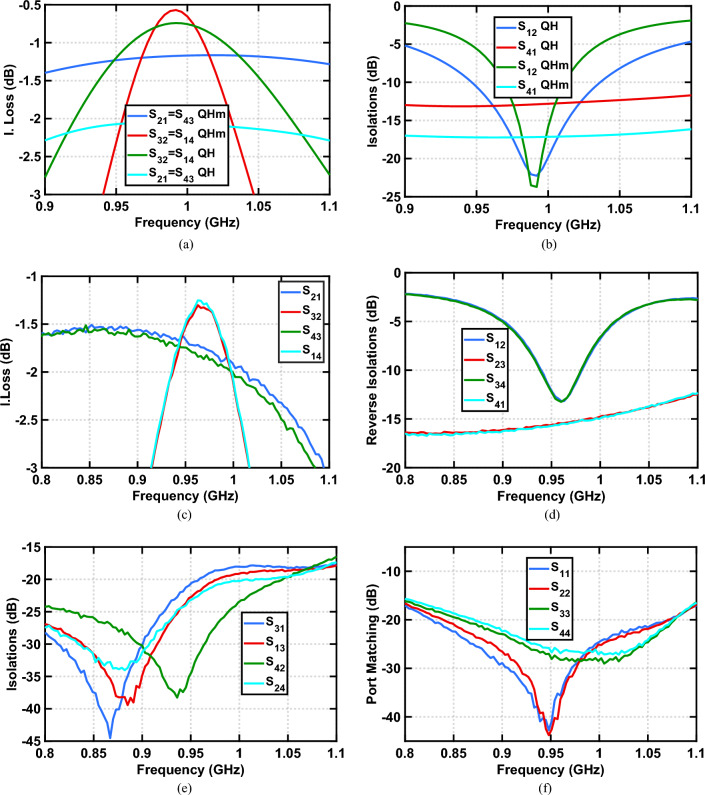
(**a**) Simulated insertion losses of four-port circulators with standard ($S_{ij} QH$) and modified ($S_{ij} QHm$) quadrature hybrids respectively. The transmission line arms of the hybrids centered at 1 GHz are implemented as ideal lumped LCL transformers with $Z_0$ values of L $=$ 8.7 nH and C $=$ 2.9 pF and $Z_0/ \sqrt{2}$ values of L $=$ 6.2 nH and C $=$ 4.1 pF. The $90^\circ$ NRPS is implemented with a 65 nm CMOS 4-path which employs a 12.5 pF baseband capacitance. (**b**) Simulated reverse isolations of the two circulators. (**c**) Measured insertion losses of a four-port circulator implemented on a Rogers RO4350 10 mil dual-sided PCB. The LCL phase shifters, comprised discrete inductors of L $=$ 8.2 nH and capacitors connected to the ground of C=2.8 pF. (**d**) Measured reverse isolation. (**e**) Measured isolation. (**f**) Measured port matching.

Employing the neutralization property on an FPEC, with the modified quadrature hybrid $S_{QHm}$, forms a QCQH that includes 3 TLs and the NRPS as shown in Fig. 1f. This QCQH is connected to ports 3 and 4 through TLs. An input signal at port 1 of a QCQH introduces a shorted NRPS to ground similar to Fig. 3b^23^. Hence, $S_{QH}$ and $S_{QHm}$ solutions impose virtual grounds at the NRPS terminals when excited from port 1. Simulation results for $S_{21}$ insertion loss in Fig. 6a with $S_{QHm}$ of (13) are 0.8 dB better than those of $S_{QH}$ of (8). We attribute the loss difference to parallel loading that each quadrature hybrid imposes at ports 1 and 2 for an input signal at port 1. In order to explain this loss difference, we assume the short to ground at the terminals of the NRPS, as depicted in Fig. 3b is practically a 5 $\Omega$ resistor to ground and utilize the $90^\circ$ TL impedance transformation property $Z_1 \cdot Z_3={Z_2}^2$; then, for the TLs that connect ports 1 and 2 to the NRPS, one can calculate a parallel loading of 500 $\Omega$ for the $S_{QHm}$ design and 250 $\Omega$ for the $S_{QH}$-this difference in parallel loading results in an insertion loss difference. The bandwidth of both solutions is relatively wide since the signal propagates between $Z_0$ ports 1 and 2 through a $Z_0$ TL for both hybrid solutions, performing no impedance transformation.

Simulations of $S_{32}$ result in a narrower bandwidth compared with $S_{21}$ as depicted in Fig. 6a. To explain this, we use the equivalent circuit shown in Fig. 3c that employs the quadrature hybrid $S_{QH}$. The signal propagating between ports 2 and 3 divides equally at the terminals of the NRPS and recombines at port 3. This path through the two hybrids is narrower in bandwidth than $S_{21}$ since the four-arm design utilized to implement $S_{QH}$ is narrowband. The simulated FPEC $S_{32}$ insertion loss for $S_{QHm}$ of (13) is 0.2 dB better at the center frequency yet has a narrower bandwidth.

The $S_{41}$ isolation in Fig. 6b for $S_{QHm}$ is 4 dB better than for $S_{QH}$ and $S_{12}$, of the first, is better at the center frequency, yet has a narrower bandwidth.

### Circuit implementation details

The FPEC, shown in Fig. 5c, was implemented on a Rogers RO4350 10 mil dual-sided PCB. The implemented $90^\circ$ LCL phase shifters, as shown in Fig. 5b, included discrete inductors of L $=$ 8.2 nH and capacitors connected to the ground of C $=$ 2.8 pF, realizing a $Z_0$ characteristic impedance and $90^\circ$ phase shifting at 1 GHz. The chip side (top) comprised the 4-path NRPS die shown in Fig. 5c with the schematic shown in Fig. 5a and a neutralized modified quadrature hybrid with three arms as in Fig. 5b. Ports 1 and 2 were connected at the top PCB side, and the terminals of the N-path joined the bottom PCB through plated via holes. The bottom PCB comprised a modified LCL quadrature hybrid. Ports 3 and 4 were placed at the far end of the bottom PCB. Schematically, the top PCB realized a QCQH^23^ and the bottom a modified hybrid. The $90^\circ$ NRPS chip microphotograph is in Fig. 5c. The total area of the chip is 740 $\mu$m $\times \,790 \mu$m, with an active area of 200 $\mu$m $\times \,600 \mu$m. The die was mounted on the PCB, and pads were connected by wire bonds, as shown in Fig. 5c.

### Measured results

The FPEC in Fig. 5 was measured and the results are shown in Fig. 6c,d. In comparison to the simulations that assumed ideal LCL phase shifters, the insertion losses shown in Fig. 6c are shifted down in frequency as a result of the practical LCL parasitic and their impact on the NRPS performance. The LCL parasitic add about 0.4–0.6 dB loss compared with the simulations in Fig. 6a. Reverse isolation measurements in Fig. 6d are in good agreement with the simulations shown in Fig. 6b. The isolation measurements between ports 1 and 3 as well as 2 and 4 are in Fig. 6e and show better than 18 dB across the frequency band of 0.8–1.1 GHz. Port matching in Fig. 6f is better than 18 dB spanning 0.8–1.1 GHz.

## Conclusions

This work presents the analysis and design of balanced chip scale four-port electronic circulators based on a $90^\circ$ NRPS connected across the symmetry line of two back-to-back quadrature hybrids. We propose a unified transfer function analysis using the three, four-port, cascaded sub-circuits. This technique also applies to four-port duplexers with only two cascaded sub-circuits and can be extended to more than three sub-circuits. Furthermore, three new NRPS properties are introduced and proved mathematically. One of these properties indicates the neutralization of the load that $90^\circ$ transmission lines impose on the NRPS when connected across its terminals. We indicate that cascaded sub-circuit analysis and the neutralization property govern all physical implementations of four-port non-reciprocal duplexers.

Synthesis of the circulators’ generalized cascaded transfer function enables the extraction of different solutions. We analyze and compare two circuit realizations of the solutions utilizing a practical CMOS NRPS. Future research may benefit the remaining physical FPEC equation solutions. Their circuit implementations may perform better with various $90^\circ$ NRPS designs. Moreover, synthesis of unified transfer functions theoretically enables the research and discovery of physical implementations of new four-port duplexers with new non-reciprocal transfer functions. One may find interest in delving into such options in future work.

## Methods

### Analysis of the $90^\circ$ NRPS Neutralization Property

Proving the virtual neutralization of a $90^\circ$ TL (PS) across an ideal $90^\circ$ NRPS requires the Y-parameter matrix of (1), which has no inverse matrix and therefore no Y definition^31^. To enable a defined Y matrix, one can connect a low-value resistor *r* in series with each terminal where the characteristic impedance of the terminals is $Z_0$. The resistors inflict reflection coefficients of $\varepsilon = r/(Z_0 +r)$ and an insertion loss factor of 1-$\varepsilon$, where $\varepsilon$, which denotes the loss factor, is positive and tends to zero with *r*. The NRPS with the resistive losses is denoted as $S_\text {N90-P}$ and is given by

14 $$\begin{aligned} S_\text {N90-P} = \left[ \begin{array}{cc} \varepsilon &{} \quad (1-\varepsilon )e^{-j{\frac{\pi }{2}}} \\ (1-\varepsilon )e^{j{\frac{\pi }{2}}} &{} \quad \varepsilon \\ \end{array} \right] \end{aligned}$$

$Y_\text {N90}$ denotes the Y-parameter form of $S_\text {N90-P}$, and can be derived from (14) employing^31^ as

15 $$\begin{aligned} Y_\text {N90} = \frac{1-\varepsilon }{2 \varepsilon } \left[ \begin{array}{cc} 1 &{} \quad e^{j\frac{\pi }{2}} \\ e^{-j\frac{\pi }{2}} &{} \quad 1 \\ \end{array} \right] \end{aligned}$$

which reveals admittance coefficients that tend to infinity when $\varepsilon$ tend to zero.

The Y-parameters of an ideal $90^\circ$ $Z_0$ transmission line are

16 $$\begin{aligned} Y_\text {TL90} = \left[ \begin{array}{cc} 0 &{} \quad e^{j\frac{\pi }{2}}\\ e^{j\frac{\pi }{2}} &{} \quad 0 \\ \end{array} \right] \end{aligned}$$

One can calculate the overall Y-parameters of the $90^\circ$ NRPS with a $90^\circ$ TL connected across its terminals by adding the Y parameters of the two elements from (15) and (16) as

17 $$\begin{aligned} Y_{{\textrm{N90}} \parallel {\textrm{TL90}}} = Y_\text {N90} + Y_\text {TL90} = \left[ \begin{array}{cc} \frac{1-\varepsilon }{2 \varepsilon } &{} \quad \frac{1+\varepsilon }{2 \varepsilon } e^{j\frac{\pi }{2}} \\ \frac{1-3 \varepsilon }{2 \varepsilon } e^{-j\frac{\pi }{2}} &{} \quad \frac{1-\varepsilon }{2 \varepsilon }\\ \end{array} \right] \end{aligned}$$

The overall S-parameters of the parallel elements are calculated from the overall Y-parameters of (17) utilizing^31^, and results in

18 $$\begin{aligned} S_{{\textrm{N90}} \parallel {\textrm{TL90}}}= \left[ \begin{array}{cc} 0 &{} \quad e^{-j{\frac{\pi }{2}}} \\ \frac{1-3\varepsilon }{1+\varepsilon }e^{j{\frac{\pi }{2}}} &{} \quad 0 \\ \end{array} \right] \underset{\varepsilon \rightarrow 0}{\approx } \left[ \begin{array}{cc} 0 &{} \quad e^{-j{\frac{\pi }{2}}} \\ e^{j{\frac{\pi }{2}}} &{} \quad 0 \\ \end{array} \right] \hspace{5mm} \end{aligned}$$

By letting $\varepsilon \rightarrow 0$, the final result is identical to (1); hence, the TL’s loading across the NRPS is neutralized. Similarly, two NRPS circuits in parallel have the same transfer function as a single NRPS. One can derive this property by summing two $Y_{N90}$ matrices from (15), converting the resulting Y to S and letting $\varepsilon$ tend to zero in order to achieve (1).

### An even-odd analysis of an FPEC

Separating the NRPS in the schematic of Fig. 3a into two parallel NRPS circuits, we create two identical circuits connected back to back across the line of symmetry. Each of these circuits includes a quadrature hybrid described by the matrix $S_{QH}$ as shown in Fig. 4b, which excites an NRPS. An even-odd analysis for an input signal at port 1 utilizes the symmetry with port 3. An even excitation disconnects the two mirrored circuits, and an odd excitation shorts them to ground at the line of symmetry across the NRPS terminals. Applying the TL neutralization property on both circuits results in the formation of two back-to-back circuits introduced and analyzed in^36^. One can utilize the transfer functions derived in^36^ for even-odd excitation to complete the analysis on each side of the line of FPEC symmetry. The same analysis applies to input signals at port 2, utilizing the symmetry with port 4 for even-odd excitation. The analysis results in (9), as expected. Moreover, this analysis applies to the modified quadrature hybrid shown in Fig. 4c described by $S_{QHm}$, utilizing the transfer functions of the QCQH in^23^.

### Cascaded sub-circuit analysis of an FPEC

The calculation of the circulator matrix $S_{FPEC}$ as in (9) for the quadrature hybrids $S_{QH}$ given in (8) and the NRPS $S_{4PortN90}$ of (11) follows (10), and the 4-port NRPS transfer function (T-matrix). Nevertheless, the transfer matrix of (11) is not defined^33,34^. Hence, as in (14), a resistive imperfection is assumed in series with the ports to enable a T form for the NRPS. Reusing $\varepsilon$, the loss factor definition from (14), the practical four-port S-matrix of the NRPS denotes as $S_\text {4PortN90-P}$ is given by

19 $$\begin{aligned} S_{4PortN90-P} = \frac{1}{2} \left[ \begin{matrix} -g &{} \quad -jg &{} \quad h &{} \quad -jg \\ jg &{} \quad -g &{} \quad jg &{} \quad h \\ h &{} \quad -jg &{} \quad -g &{} \quad -jg \\ jg &{} \quad h &{} \quad jg &{} \quad -g \end{matrix} \right] \end{aligned}$$

where

20 $$\begin{aligned} g=\frac{1-\varepsilon }{1+\varepsilon }\hspace{2mm}, \hspace{5mm} h=\frac{1+\varepsilon }{1-\varepsilon } \end{aligned}$$

Since $h^2-g^2 \ne 0$, the T-form of the practical four-port NRPS in (19), $T_\text {4PortN90-P}$, is defined^33,34^.

The transfer function $T_\text {FPEC}$ is calculated from (10) utilizing the T matrices of (8) and (19). The result of (10) is converted to S-parameters as in^33,34^ and the final matrix $S_\text {FPEC}$ is obtained by letting $\varepsilon \rightarrow 0$ and is identical to (9). This analysis applies to the modified quadrature hybrid described by $S_{QHm}$ of (13) as well.

### Cascaded sub-circuit analysis of the three-port circulator

To demonstrate the scalability of the analysis approach presented in this paper, we analyze the fundamental topology of the three-port circulator of^19,20^ and^21^. Utilizing Fig. 4a and following the derivation of (11), a similar four-port transfer function can be derived for the case when port 3 of the NRPS is open. The respective S-matrix is given by

21 $$\begin{aligned} S_\text {3PortN90} = \frac{1}{3} \left[ \begin{matrix} -1 &{} \quad -2j &{} \quad 0 &{} \quad -2j \\ 2j &{} \quad -1 &{} \quad 0 &{} \quad 2 \\ 0 &{} \quad 0 &{} \quad 3 &{} \quad 0 \\ 2j &{} \quad 2 &{} \quad 0 &{} \quad -1 \end{matrix} \right] \end{aligned}$$

It is simple to verify that cascading (13) with (21) achieves the four-port representation of the three-port circulator as given in^23^, eq. 8. Similarly, one can employ (11) or (19) analyzing or synthesizing topologies with more than three-cascaded sub-circuits.

### Derivation of the general FPEC solution

The solution $S_{QHn}$ in (12) represents a passive network, and must be reciprocal fulfilling $S_{ij}=S_{ji}$. Direct optimizations on the general FPEC circuit shown in Fig. 3a, utilizing the S-matrices $S_{FPEC}$ in (9) and $S_{4PortN90}$ in (11), reveal $S_{QHn}$ solutions in the form of (12), where a, b, c and d can be real as in (8) and (13). Solutions comprising complex coefficients exist as well.

Alternatively, direct analytical computations may be used. The general form of (10), for the circuit shown in Fig. 3a, is written as

22 $$\begin{aligned} T_\text {FPEC} = T_{QHn} \cdot T_\text {4PortN90} \cdot T_{QHn} \end{aligned}$$

where $T_{QHn}$ is the free variable describing the transfer matrix of the passive networks of the FPEC as shown in Fig. 3a. Substituting the respective T forms $B=T_{FPEC}$, $A=T_{4PortN90}$ of (19), we can calculate $X=T_{QHn}$ by solving $B=X\cdot A\cdot X$. One can show that if *AB* is diagonalizable and *A* is invertible, then sixteen mathematical solutions exist in theory. Since B, the T-matrix of the ideal FPEC in (9) is undefined^33,34^, a practical S-matrix denoted as $S_\text {FPEC-P}$ that includes resistive losses in series with every port serves in the general solution of (22). The matrix $S_\text {FPEC-P}$ enables an invertible $T_\text {FPEC}$ and is given by

23 $$\begin{aligned} S_{FPEC-P} = \left[ \begin{matrix} k &{} \quad l &{} \quad o &{} \quad jp \\ -jp &{} \quad k &{} \quad l &{} \quad o \\ o &{} \quad jp &{} \quad k &{} \quad l \\ l &{} \quad o &{} \quad -jp &{} \quad k \\ \end{matrix} \right] \end{aligned}$$

where

24 $$\begin{aligned} k \approx \varepsilon _1\hspace{0.2mm}, \hspace{1.8mm} l \approx \varepsilon _1 ^2 \hspace{0.2mm}, \hspace{1.8mm} o \approx \varepsilon _1 - 2 \varepsilon _1 ^2 \hspace{0.2mm}, \hspace{1.8mm} p \approx 1-2 \varepsilon _1 + \varepsilon _1 ^2 \end{aligned}$$

The loss factor, $\varepsilon _1$ in this case, is different from $\varepsilon$ and given by $\varepsilon _1 = r/(2 Z_0 +r)$, where *r* is the low-value resistance added at each port.

The general solution for X is derived by multiplying (22) by A and solving

25 $$\begin{aligned} A \cdot B = A\cdot X \cdot A\cdot X=(A\cdot X)^2 \end{aligned}$$

and result in $X=A^{-1}\cdot (A\cdot B)^{1/2}$.

Upon converting the $T_\text {FPEC}$ result to S-parameters following the conversion procedure outlined in^33,34^ and taking the limit $r \rightarrow 0$, the extracted general solutions are centrosymmetric matrices, with a diagonal symmetry adhering to $S_{I,I}=S_{II,II}$ and $S_{II,I}=S_{I,II}$^33,34^.

Assuming physical lossless solutions, one can write

26 $$\begin{aligned} |S_{1j}|^2+|S_{2j}|^2+|S_{3j}|^2+|S_{4j}|^2 = 1 \end{aligned}$$

Solutions of (22) which do not adhere to (26) are not physical. One such example utilizes the complex coefficients $a_c$ = 0.237-0.304j, $b_c$ = 0.448-j, $c_c$ = 0.31–0.116j and $d_c$ = 0.776-0.531j which are a solution to (22) but do not conform to (26).

### Measurement setup

A differential clock signal of 8 dBm at 2 GHz is derived from a signal generator through a $180^\circ$ hybrid coupler to the chips’ clock inputs. The signal frequency is divided by 2 on-chip to generate the four clock phases needed for the 4-path circuit utilized as $90^\circ$ NRPS. The chip is biased from a 1.2-volt power supply, and the total power consumption at 1 GHz operation is 15 mW. A two-port vector network analyzer serves in the S-parameter measurements of the circulator. Six two-port tests yield the total four-port performance, whereas the ports not under test are terminated with broadband 50 $\Omega$ loads.

## Data Availability

The datasets used and/or analysed during the current study available from the corresponding author on reasonable request.
